# Brain Cortical Thickness Differences in Adolescent Females with Substance Use Disorders

**DOI:** 10.1371/journal.pone.0152983

**Published:** 2016-04-06

**Authors:** Peter K. Boulos, Manish S. Dalwani, Jody Tanabe, Susan K. Mikulich-Gilbertson, Marie T. Banich, Thomas J. Crowley, Joseph T. Sakai

**Affiliations:** 1 University of Colorado Denver School of Medicine, Aurora, Colorado, United States of America; 2 Division of Substance Dependence, Department of Psychiatry, University of Colorado Denver School of Medicine, Aurora, Colorado, United States of America; 3 Department of Radiology, University of Colorado Denver School of Medicine, Aurora, Colorado, United States of America; 4 Institute of Cognitive Science, Department of Psychology and Neuroscience, University of Colorado Boulder, Boulder, Colorado, United States of America; Banner Alzheimer's Institute, UNITED STATES

## Abstract

Some youths develop multiple substance use disorders early in adolescence and have severe, persistent courses. Such youths often exhibit impulsivity, risk-taking, and problems of inhibition. However, relatively little is known about the possible brain bases of these behavioral traits, especially among females.

**Methods:**

We recruited right-handed female patients, 14–19 years of age, from a university-based treatment program for youths with substance use disorders and community controls similar for age, race and zip code of residence. We obtained 43 T1-weighted structural brain images (22 patients and 21 controls) to examine group differences in cortical thickness across the entire brain as well as six *a priori* regions-of-interest: 1) medial orbitofrontal cortex; 2) rostral anterior cingulate cortex; and 3) middle frontal cortex, in each hemisphere. Age and IQ were entered as nuisance factors for all analyses.

**Results:**

*A priori* region-of-interest analyses yielded no significant differences. However, whole-brain group comparisons revealed that the left pregenual rostral anterior cingulate cortex extending into the left medial orbitofrontal region (355.84 mm^2^ in size), a subset of two of our *a priori* regions-of-interest, was significantly thinner in patients compared to controls (vertex-level threshold p = 0.005 and cluster-level family wise error corrected threshold p = 0.05). The whole-brain group differences did not survive after adjusting for depression or externalizing scores. Whole-brain within-patient analyses demonstrated a positive association between cortical thickness in the left precuneus and behavioral disinhibition scores (458.23 mm^2^ in size).

**Conclusions:**

Adolescent females with substance use disorders have significant differences in brain cortical thickness in regions engaged by the default mode network and that have been associated with problems of emotional dysregulation, inhibition, and behavioral control in past studies.

## Introduction

Some individuals have onset of substance use disorder (SUD) early in adolescence, develop multiple SUD diagnoses and have severe persistent courses [1, 2]. These youth exhibit problems of self-control and risk taking in real life and laboratory settings [2–4] and such problems of inhibition may stem from measurable brain differences. Brain structural differences associated with these behavioral phenotypes are poorly understood in females. Therefore, we tested whether adolescent females with early onset substance use problems differ from controls in brain cortical thickness.

### A Focus on Youths with Child/Adolescent-Onset Substance Use Problems

Despite important and recent advances [5], our understanding of the neurobiology of SUDs remains insufficient. SUDs are common in the general population [6], are a source of great morbidity and mortality [6, 7], and exact a huge cost to society in drug-related crime, health care costs, and productivity losses [8]. Although many youths experiment with substances [9], most will not progress to develop a SUD [6]. While it is well documented that these disorders cluster within families [10] and are moderately heritable [11–14], it is not soundly understood at a biological level why some youth appear more prone to develop a SUD.

Considering those who develop a SUD, the peak age of onset is in later adolescence or young adulthood, with less common onset after age of 25 [15]. However, some individuals have onset of SUD early in adolescence, develop multiple SUD diagnoses, and have severe persistent courses [1, 2]. Youths in this population are likely to have a number of precursors, characteristic co-morbidities, and associated cognitive deficits. For example, youths with poor self-control [16], low constraint [17], and early problems with inhibition [18, 19] are at an increased risk for later developing SUDs. Youths with SUDs also display risk-taking [3], impulsivity [20], difficulty delaying gratification [21], and impaired performance on laboratory cognitive tasks [22, 23]. Youths of both genders with SUDs are also very likely to have co-morbid conduct disorder [24, 25] and individuals with conduct disorder on average initiate substance use at an early age [26]. While conduct disorder is more common in males, it is still prevalent in adolescent girls, representing the second most common psychiatric diagnosis in female adolescents [27]. This clustering of high externalizing behavior problems within individuals with SUD is sometimes referred to as behavioral disinhibition (BD), a highly heritable (h^2^>0.8) latent trait [11,14, 19, 28] which has a strong genetic correlation with laboratory-measured problems of executive control [29].

### A Female-Only Sample

Adolescence is a time in which many sex differences begin to emerge with regards to psychopathology (e.g., rates of depression; [30]) and these sex differences appear to be mirrored by sex differences in brain development [31, 32]. Becker et al. review the broad literature of neural networks mediating addiction, highlighting clear sex-differences in dopaminergic, noradrenergic, corticotropic, opioid, and cholinergic pathways. The authors suggest that these differences may correlate with distinct clinical presentations of addiction in females and emphasize the importance of studying males and females separately [33]. Hardee et al. recently presented their findings from a longitudinal fMRI study showing clear differences between males and females in amygdala and premotor cortex activation, in support of the proposition that the development of SUD in females is more likely to be related to negative affectivity, whereas in males, risk may be more likely mediated by impulsiveness [34]. While there has been some examination of differences in behavior and brain anatomy/function in boys with and without SUD or related phenotypes [35–43], little research has examined girls [36, 44]. Behaviorally, SUD in girls looks somewhat different than boys. For example, although the prevalence of substance use is similar between young adolescent males and females, with increasing age a higher SUD prevalence develops in males [6]. Females also show telescoping effects, having faster rates of progression from use to dependence, resulting in more severe clinical profiles upon presentation to treatment despite less or equivalent total substance use [45]. Other studies of SUD suggest male-female differences in genetic contributors [46] and environmental risks [47].

Considering these differences in behavior, and the clear sex differences in brain anatomy/function during adolescence, it is reasonable that males and females may have different, as well as overlapping, biological underpinnings to SUD. As anatomical differences between boys with and without SUD have been clearly documented, the current paper focuses on anatomy in females with and without SUD. In addition, given the literature on externalizing problems and, especially in females, affect regulation, we also seek here to explore whether patient-control differences covary with the severity of these comorbidities.

### Brain Cortical Thickness

Several brain regions have been implicated in *volumetric* studies of youths with serious SUD, youths with high BD, or similar phenotypes. These include the insula [35, 36], dorsolateral prefrontal cortex (DLPFC) [37], orbitofrontal cortex (OFC) [38], and anterior cingulate cortex (ACC) [39–43], among others. This literature of volumetric studies is rapidly growing but, to our knowledge, few of these studies have focused on adolescent females specifically [36, 44]. In addition, relatively few studies of cerebral *cortical thickness* have been previously conducted on these adolescent phenotypes. Adolescent heavy marijuana users reportedly have cortical thinning in right caudal middle frontal regions, bilateral insula, and bilateral superior frontal cortex along with increased cortical thickness in the lingual, superior temporal, inferior parietal and paracentral regions [48]. Adolescents with "gaming addiction" [49] and "internet addiction" [50] have shown cortical thinning in the orbitofrontal cortex and elsewhere. However, to our knowledge, none of these previous studies focus on cortical thickness in female-only samples. Instead most prior studies have used male-only or mixed-sex samples. Although the literature on cortical thickness is more limited, available volumetric studies strongly suggest that prefrontal cortex, including the ACC, DLPFC, and OFC sub-regions are involved in SUD. These regions participate in behavior inhibition, executive functions, and decision-making [51]; localized lesions in these regions are associated with significant impairment in neuropsychological function, similar to those discussed with BD patients [52]. Thus, the ACC, DLPFC, and OFC are logical targets for region-of-interest analyses.

While volume and thickness are related, they are distinct phenotypes. According to the radial unit hypothesis, cells with the same origin are organized into columns, which run perpendicular to the brain’s surface [53–56]. The number of columns determines surface area, which is strongly related to grey matter volume, while the number of cells within a column determines cortical thickness [57, 58]. Both surface area and cortical thickness are heritable but available twin work supports that they have different genetic determinants [57, 59]. Thus, studying cortical thickness as we do here provides important complementary information to our recently published volumetric work [60].

### Study Hypothesis

We hypothesized that whole-brain and region-of-interest analyses would identify differences in cortical thickness in prefrontal (especially anterior cingulate, middle frontal gyrus and orbitofrontal cortex) brain regions in female adolescents with early onset SUD, compared to controls.

## Methods

The Colorado Multiple Institutions Review Board (COMIRB) approved all procedures and the study consents. Subjects 18 years of age or older provided written consent; those under 18 provided written assent while their parents provided written consent to study participation. Different data from this sample are reported in Dalwani et al., (volumetric results) [60] and Crowley et al., (fMRI data results) [61].

### Sample

We report on 22 patients and 21 controls. All were female, aged 14–19 years, had an estimated IQ ≥ 80, and adequate English proficiency to understand the study consents.

Patients were recruited from a university-based adolescent treatment program for youths with serious substance and conduct problems and were required to meet criteria for at least one non-nicotine DSM-IV-TR substance abuse or dependence diagnosis [62]. To reduce confounds from intoxication or recent drug use, we required patients to have multiple negative urine (AccuTest^™^ for THC, cocaine, methamphetamine, amphetamine, barbiturates, benzodiazepines, MDMA, methadone, other opioids, PCP) and saliva (AlcoScreen^™^ for alcohol) tests for at least 7 days prior to scanning. 26 patients were enrolled in the study but did not complete MRI scanning for reasons including positive urinalysis, not meeting substance use disorder screening criteria, IQ < 80, MRI contraindications, epilepsy, positive pregnancy test, court-ordered ankle monitor that could not be removed, or simply no longer willing to participate.

Controls, contacted first by advertising or by a research marketing company, were similar to the patient group with respect to age, race, and zip code of residence. Exclusion criteria for controls included previous court conviction (excluding minor traffic or curfew offenses), a substance-related arrest or treatment, school expulsions, meeting DSM-IV-TR criteria for a non-nicotine substance abuse or dependence diagnosis, meeting DSM-IV-TR criteria for conduct disorder in the last year, or a positive test for a non-prescribed substance about 7 days before and immediately prior to scanning using the same urine and saliva tests mentioned above. Four controls were enrolled in the study but did not complete MRI scanning for reasons including IQ < 80 or meeting criteria for a non-nicotine SUD

For all subjects, we applied standard MRI exclusion criteria (e.g. orthodontic braces, claustrophobia, ferric metal in the body) for adolescents. Subjects with a positive pregnancy test, history of serious neurological illness, prior neurosurgery, or a history of unconsciousness lasting greater than 15 minutes were also excluded. Because the scanning session also acquired fMRI data using a paradigm requiring subjects to distinguish green from red for use in another study, color blindness was an additional exclusion criterion. Prior work showing cortical asymmetry amongst right- and left-handed individuals resulted in the exclusion of left-handed adolescents from these analyses [63, 64]. Exclusion criteria for all subjects also included current high risk of suicide, psychosis, violence, or fire setting.

### Assessments

Each participant completed numerous psychosocial assessments before MRI scanning [65]. Parents of each adolescent completed the Child Behavior Checklist (CBCL) and an updated Hollingshead Four-Factor Index of socioeconomic status [66]. The CBCL assessed attention-deficit/hyperactivity disorder (ADHD) symptoms and associated problems [67]. Each adolescent completed the vocabulary and matrix reasoning subtests of the Wechsler Abbreviated Scale of Intelligence to provide an estimate of IQ [68], the Youth Self Report (YSR), the Composite International Diagnostic Interview—Substance Abuse Module (CIDI-SAM), the Diagnostic Interview Schedule for Children (DISC), a Peak Aggression Scale (PAS) [2], the Eysenck Junior Impulsiveness Scale (EJIS) for a measure of impulsivity [69], and finally the Carroll Rating Scale for Depression (CRS). The YSR measure of ADHD symptoms was substituted for those participants (n = 6, all in the patient group) that did not have a CBCL available [70]. The CIDI-SAM and supplement served to generate DSM-IV SUD diagnoses and to determine recency of substance use, respectively [71]. From CIDI-SAM data, we also calculated SUMDEP, the total number of substance dependence symptoms across 10 different categories (range 0 to 70). We have used this measure in previous studies to compare groups on substance use severity [2]. The DISC assessed lifetime DSM-IV conduct disorder diagnoses [72] and the CRS estimated depression scores [73]. These assessments were completed in one session lasting approximately 3 hours.

Behavioral disinhibition (BD) scores combined information from 4 behavioral measures: DSM-IV symptom counts for conduct disorder, CBCL/YSR-measured scores of inattention (questions 8, 13,17, 61, and 80) and impulsivity (questions 1, 10, 36, 41, 45, 46, 62, 93, and 104), and sum of abuse/dependence symptoms across 10 drug categories. Subjects’ scores were normed to a community sample of 414 adolescent females (i.e. number of standard deviations from the community sample mean). Utilizing this community sample, principal component analyses extracted the maximum covariance among the 4 behavioral measures and the resulting standardized factor loadings (on the first principal component) were utilized to weight our 4 standardized behavioral measures and sum them to generate BD scores (see http://ibgwww.colorado.edu/cadd/bd.html for details; [61]). We chose this validated measure of externalizing behavior, as opposed to other broader measures, as it takes into account those specific externalizing traits commonly comorbid with SUD (see Introduction, A Focus on Youths with Child/Adolescent-Onset Substance Use Problems).

### MRI Parameters

A General Electric 3T MRI scanner was used to acquire high-resolution 3D T1-weighted images, taken along the coronal plane, using an SPGR-IR sequence and a standard quadrature head coil. The parameters were: TR = 9 ms, TE = 1.9 ms, T1 = 500 ms, flip angle = 10°, FOV = 220 mm^2^, slice thickness = 1.7 mm, and matrix = 256x256, 0.97 x 0.97 mm^2^ in plane. Scan time was 9 minutes and 12 seconds to acquire 124 slices.

### Data Analyses

We compared groups for differences in demographic (e.g. age, race, SES) and diagnostic data (e.g. attention-deficit/hyperactivity disorder, conduct disorder, substance use disorder diagnoses) using SPSS software (IBM SPSS Statistics, Version 21. Chicago, IL: IBM Corp; 2012). Chi-square or Fisher’s Exact tests were used to compare categorical variables and t-tests or Mann-Whitney U tests were appropriately performed for continuous variables. We conducted all of these analyses using two-tailed tests at a 0.05 significance level.

### FreeSurfer Analyses

MRI scans were reconstructed to measure cortical thickness using FreeSurfer software version 5.3.0. FreeSurfer reconstructs the images by first fitting the image to Talairach space, stripping non-brain structures from the image, forming the outermost grey matter boundary, and finally forming a white/grey matter boundary. The program utilizes triangular tessellation and surface deformation algorithms to form the boundaries. Cortical thickness is measured as the distance from the outer grey boundary, the pial surface, to the white/grey boundary [74]. A single team member blinded to the subjects’ group affiliation ensured that the software performed the reconstructions properly by conducting a slice-by-slice visual inspection of each step of the reconstruction for all subjects in 3 planes (coronal, sagittal, and horizontal). As needed, edits were performed consistently throughout the sample and then the edited images were run through the program again. Necessary edits included: ensuring proper fit into Talairach space, manually stripping skull that the program missed, and adding control points to areas that were assuredly white matter but were not appropriately recognized as white matter. The temporal lobe commonly demanded edits. The effects of these edits on the results were examined (See S1 Text. Testing the Effects of Edits). The program then automatically parfcellated the reconstructed brain into regions according to Desikan’s atlas [75].

### Brain Morphometry Analysis

We conducted whole-brain and region-of-interest (ROI) analyses. The whole-brain analysis was performed using FreeSurfer’s QDEC program while adjusting for age and IQ by entering them as nuisance factors. QDEC smoothed the data with a 10 mm full width at half maximum Gaussian kernel, while enforcing a Monte Carlo cluster correction (250 mm^2^) with a vertex-level threshold of p < 0.005. SPSS was used to conduct the ROI analyses on extracted regions. We examined 3 ROIs bilaterally (total of 6 ROIs) defined by the Desikan’s atlas [75] for our *a priori* predictions based on published literature (see Introduction, Brain Cortical Thickness, paragraph 1). These regions were: 1) medial orbitofrontal cortex (mOFC); 2) rostral anterior cingulate cortex (RACC); 3) middle frontal gyrus (MFG). In order to calculate MFG cortical thickness we combined surface-area-adjusted values for rostral middle frontal cortex and caudal middle frontal cortex as measured according to the Desikan’s atlas. Regression analyses tested for group differences while controlling for age and IQ for each ROI. This approach to perform both whole-brain and *a priori* identified ROI analyses follows procedures used in past studies [31, 48].

### Secondary Analyses

#### Patient-only regression analyses

We explored differences among patients that affect cortical thickness. To do this, we conducted within-patient regression analyses examining association of cortical thickness with recency of drug use (a single variable calculated from number of days since last use of any non-tobacco substance) and separately with severity of BD after adjusting for age and IQ. This was done as both a whole-brain vertex-level analysis and also utilizing a virtual mask to include only those regions that differed significantly in patient-control comparisons.

#### Testing how patient-control cortical thickness differences relate to differences in internalizing and externalizing behavior problems

To investigate how differences in cortical thickness between patients and controls might relate to internalizing and externalizing measures we performed additional QDEC analyses using the same procedures as for our primary whole-brain analysis (described in section 2.6), with the same Monte Carlo cluster correction (250 mm^2^) and vertex-level threshold (p < 0.005). In addition to age and IQ, we evaluated depression scores from the CRS, total anxiety scores from the YSR, total affectivity scores from the YSR, or total externalizing scores from the YSR as covariates in 4 separate analyses.

#### Testing for sex differences

Lastly, we have previously published very similar analyses testing brain cortical thickness patient-control differences in cortical thickness in a male adolescent sample [76]. This study used essentially the same recruitment procedures, inclusion/exclusion criteria and imaging parameters (see S1 Table. Comparing Inclusion and Exclusion Criteria for Our Female Sample with Male Sample Published Previously). Males and females from our patient, and separately control samples, were similar for demographic and clinical measures, except conduct disorder prevalence in patients (see S2 Table. Comparing Males and Females Within Patients and Within Controls for Demographics and Key Clinical Measures). Although our focus in this study is squarely on patient-control differences in a female sample, and we do not wish to duplicate reports of these previously published male patient-control findings, we utilized this male sample in these secondary analyses to explore sex differences. We therefore completed female vs male comparisons for cortical thickness differences, while controlling for age and IQ, within-patients and within-controls. Again, we used the same procedures and parameters as our primary whole-brain analysis.

## Results

### Demographic and Clinical Assessments

Table 1 compiles demographic, diagnostic, and substance use data along with other sample characteristics. There was a trend for age to differ between groups (p = 0.08) with controls being slightly older (16.67 years) than patients (16.09 years). Controls had significantly higher IQ than patients (p = 0.004; Mean IQ controls: 103.95; Mean IQ patients: 94.26). As a result we adjusted for age and IQ in all analyses. As expected, patients and controls significantly differed on various clinical measures including combined ADHD raw scores, lifetime conduct disorder diagnoses, aggression scores, impulsivity scores, and depression scores.

**Table 1:** Adolescent controls and patients: comparing demographic and diagnostic differences.

Measure	Controls (n = 21) mean(SEM) or n	Patients (n = 22) mean(SEM) or n	Test Statistic	p-value
**Demographic Data**				
Age in years	16.67 (0.25)	16.09 (0.20)	t_41_ = 1.84	0.08
Race				
Caucasian	13	12		
African American	1	1		
Hispanic	1	7		
Other	6	2		
Caucasian vs. non-Caucasian			*χ*^2^ = 0.24	0.62
Education-Highest grade completed	10.00 (0.30)	8.77 (0.17)	Mann-Whitney U	0.0021
Socioeconomic status^1^	36.14 (3.57)	45.19 (3.34)	t_35_ = 1.80	0.08
**Diagnostic Data**				
Estimated IQ	103.95 (2.26)	94.26 (2.23)	t_41_ = 3.02	0.004
Combined ADHD	1.48 (0.40)	5.68 (0.81)	t_30.60_ = -4.66	<0.001
CD lifetime diagnosis	0/21	14/22	*χ*^2^ = 19.82	<0.0001
Aggression^2^	0/21	21/22	*χ*^2^ = 39.18	<0.0001
Impulsivity	5.62 (1.00)	14.68 (1.23)	t_41_ = 5.69	<0.0001
Depression	4.33 (0.78)	10.95 (1.23)	t_35.12_ = 4.50	<0.0001
Lifetime DSM-IV-defined SUD				
Alcohol	0	19	*χ*^2^ = 32.49	<0.0001
Tobacco	0	10	Fisher’s Exact	0.0005
Cannabis	0	20	*χ*^2^ = 35.69	<0.0001
Club Drugs	0	10	Fisher’s Exact	0.0005
Cocaine	0	4	Fisher’s Exact	0.11
Hallucinogens	0	1	Fisher’s Exact	1
Amphetamine	0	4	Fisher’s Exact	0.11
SUMDEP	0.24 (0.24)	13.09 (1.66)	Mann-Whitney U	<0.0001
Length of substance dependence^3^	N/A	1.53 years (0.29)		

^1^ For 6 patients, parents did not complete questionnaires (SES and CBCL).

^2^ Note: all controls had aggression scores of 0 (mean = 0/SE = 0). Twenty-one patients had recorded aggression scores >0 (range: 1-9/mean = 5.73/SE = 0.55).

^3^ Length of substance dependence was calculated using these steps for each of the n = 20 patients meeting at least 1 substance dependence diagnosis. For one subject, considering all 10 drug categories, earliest age of substance dependence onset was subtracted from exact age at assessment.

Abbreviations: CD = conduct disorder; club drugs = ecstasy or MDMA, GHB, ketamine, rohypnol as defined by the CIDI-SAM; Combined ADHD = DSM-IV-TR defined attention-deficit/hyperactivity disorder raw scores measured using the CBCL or YSR (n = 6) if CBCL unavailable; estimated IQ = intelligence quotient estimated using the vocabulary and matrix reasoning subtests of the Wechsler Abbreviated Scale of Intelligence; SEM = standard error of the mean; SES = socioeconomic status measured using the Hollingshead Four-Factor Index; SUD = substance use disorders; SUMDEP = total number of substance dependence symptoms across 10 drug categories.

### Region-of-Interest Analysis

Female patients and controls did not differ significantly in cortical thickness in regression analyses of the 6 regions of interest while controlling for age and IQ (Left-mOFC Beta = -0.16, p = 0.37; Right-mOFC Beta = -0.07, p = 0.69; Left-RACC Beta 0.22, p = 0.24; Right-RACC Beta = -0.06, p = 0.76; Left-MFG Beta 0.09, p = 0.62; Right-MFG Beta = 0.31, p = 0.08).

### Whole-Brain Analysis

**Fig 1:**
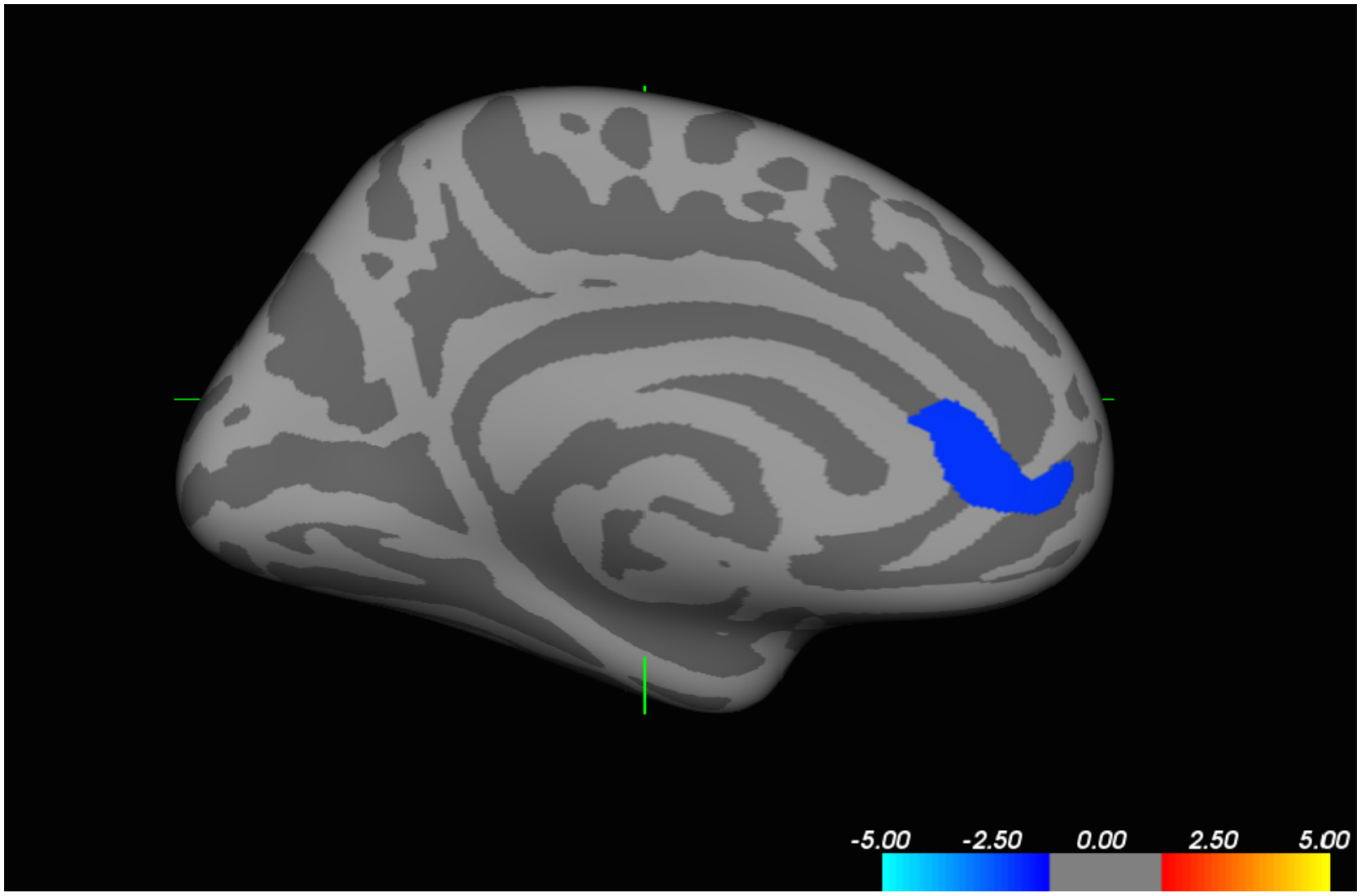
Whole-brain analyses testing for female patient-control differences in cortical thickness in QDEC using a vertex-level threshold of p<0.005 and Monte-Carlo simulation generated cluster level threshold. Medial view of left hemisphere here shows control>patient differences in cortical thickness of the pregenual rostral anterior cingulate cortex extending to the medial orbitofrontal cortex.

With specified vertex-level p < 0.005 and cluster threshold (250 mm^2^), female patients had significantly less cortical thickness than controls in left pregenual rostral anterior cingulate cortex extending into the medial orbitofrontal region, including parts of Brodmann Areas 24, 32 and 10 (MNI coordinates for center of region: x = -6.7, y = 39.5, z = 2.6; see Fig 1). The region was 355.84 mm^2^ in area and is a subset of both our RACC and mOFC *a priori* defined ROIs, but is not circumscribed by strict anatomical boundaries from either ROI.

### Secondary Analyses

#### Patient-only regression analyses

**Fig 2:**
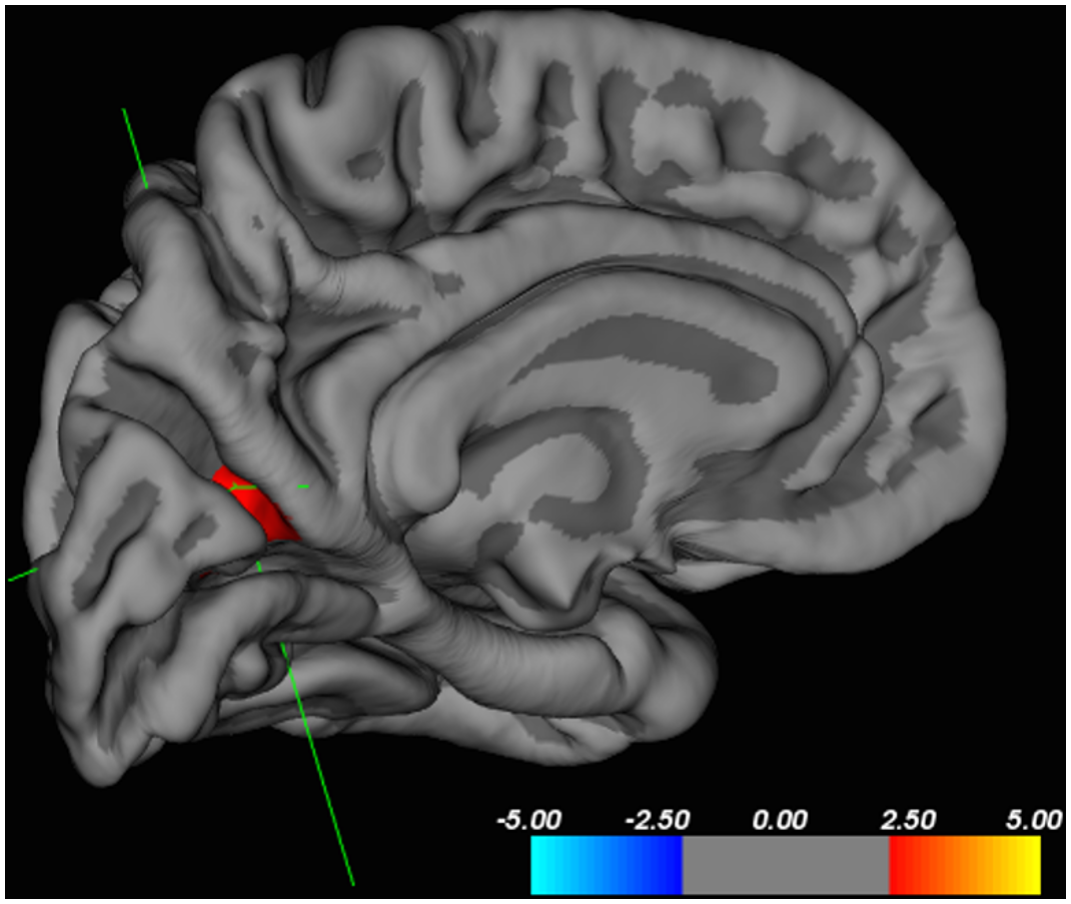
Whole-brain regression analyses within the patient group for correlation between cortical thickness and BD severity in QDEC (see Methods, Data Analyses and Discussion, ROI vs. Whole Brain Results for explanation of BD scores). Medial view of left hemisphere here shows positive correlation between BD scores and cortical thickness of the precuneus.

Regression analyses within the patient group, adjusted for age and IQ, revealed no correlation between either recency of substance use nor BD scores with cortical thickness of the RACC-mOFC cluster identified in patient-control whole-brain analyses. Performing these regression analyses on a whole-brain basis did not show any associations between cortical thickness and recency of use. However, these whole-brain regression analyses identified a positive correlation between BD severity and cortical thickness in a cluster in the left precuneus measuring 458.23 mm^2^ (MNI coordinates for center of region: x = -21.1, y = -61.3, z = 17.7; see Fig 2).

#### Testing how patient-control cortical thickness differences relate to differences in internalizing and externalizing behavior problems

The RACC-mOFC finding identified in our patient-control whole-brain analysis did not survive after additionally adjusting for either CRS depression scores or total externalizing scores from the YSR. The RACC-mOFC finding did survive, but was smaller, when additionally adjusting for either total anxiety (313.79 mm^2^) or total affectivity scores (299.86 mm^2^) from the YSR.

#### Testing for sex differences

Using our previously-published male sample [76] for comparisons, we found no differences in cortical thickness between male and female patients and no differences between male and female controls.

## Discussion

Our results suggest that adolescent females with serious substance use problems have reduced cortical thickness in pregenual regions of the left rostral anterior cingulate and medial orbitofrontal cortex (RACC-mOFC) and that left precuneus cortical thickness is positively associated with BD scores within patients.

### Current Understanding of RACC, mOFC, and Default Mode Network

The anterior cingulate and medial frontal cortices benefit from both a central anatomic location and rich interconnections with important regions and circuits, suggesting their potential importance in information processing and regulation. PET and functional MRI studies suggest an anatomical division of the anterior cingulate into rostral and caudal portions [77], with the RACC having dense connections to nucleus accumbens [78], limbic and affective systems, among other regions [43, 77]. RACC appears to play an important role in limbic regulation with respect to emotional processing and emotional conflict resolution [79, 80]. RACC is further implicated as the region primarily activated by self-referential thought and reflection [81].

A medial-lateral distinction has been proposed in OFC with the lateral OFC evaluating punishments and playing a role in response inhibition, and mOFC subserving monitoring and learning related to reinforcer valuation [82]. OFC has efferent or reciprocal connections with various brain regions including ACC, amygdala, caudate, and ventral tegmental area [83]. Through those connections, OFC may play a role in motivated behavior and assigning emotional valence to possible actions [84]. Thus together RACC and mOFC can be conceived of as an important hub, integrating sensory and visceral information, valuing reward of potential choices, and driving emotional reflection and response; these regions also play important roles in learning from errors, and engaging cognitive control regions (e.g. lateral prefrontal cortex) when necessary [79, 80, 82, 85, 86].

However, as shown in Fig 1, our RACC-mOFC cortical thickness finding from our whole-brain analyses, covers only a small portion of these regions and based on size and location appears to implicate an important hub of the Default Mode Network (DMN) [87, 88]. The DMN activates in task-free periods of rest and is characterized by functional connectivity between the posterior cingulate/precuneus, inferior parietal cortex, and the ventromedial prefrontal cortex [87]. These connections develop with age from a “local to a distributed” network. Connectivity and activation in DMN regions are seen in children as young as 8 years of age, and adolescence represents a time of DMN maturation [89]. The DMN is associated with intrinsic thought, self-reflection, and emotional processing with instructions such as, “focus on one’s feelings, one’s character, one’s memories, and one’s aspirations” activating the network [87]. Thus, the observed group differences in cortical thickness within RACC-mOFC map well to a hub of the DMN and may indicate patient-control differences in DMN, though resting state functional data would be required to confirm this.

### Relating Our Findings to Female Youths with Substance Use Disorders

Our finding of control>patient cortical thickness in the left pregenual RACC-mOFC is consistent with two complementary lines of research. First, available neuroimaging work on substance use disordered populations implicates patient-control differences in this important region. Second, available data on roles subserved by RACC and OFC fit with phenotypic descriptions of female youths with SUD.

Past MRI studies of both healthy and SUD populations point to this medial prefrontal region. For example, in normative populations, past work has suggested a link between children’s anterior cingulate volume with performance on a Go/No-Go task [43]. A negative relationship has been demonstrated between OFC cortical thickness and impulsivity in adults [90], and also between RACC cortical thickness and impulsive aggression in children [41]. Studies of SUD populations compared to controls have shown significant hypoactivity of RACC during Go/No-Go tasks [39, 40] and less grey matter concentration in mOFC and ACC, among other regions, in cocaine-dependent adults [91, 92]. Studies of grey matter volume have also linked smaller OFC volume and conduct disorder [38], though other studies implicate other frontal [37] or temporal [35] regions. Compellingly, cannabis and ecstasy users show reduced deactivation in DMN during Go/No-Go task performance compared to healthy controls indicating a failure to inhibit default-mode circuitry [93]. Thus, several lines of research link hypoactivity, less grey matter volume, and less cortical thickness within the region identified in our whole-brain analyses as affected or altered in SUD.

As described above (see Discussion, Current Understanding of RACC, mOFC, and Default Mode Network) RACC-OFC has been proposed as one important hub for integration of emotional, sensory and visceral information, aiding in valuation of expected rewards for competing potential choices. Therefore, individuals with deficits in these regions might be hypothesized to have difficulty with affective control. Relating this with our sample of adolescent females, we see a phenotypic link with emphasis placed on emotional dysregulation. Congruent with this idea, development of SUD in females is associated with negative affectivity and emotional reactivity, along with externalizing behavior problems, while in adolescent males SUD is mainly associated with externalizing problems [47]. Patients with depression have a blunted ability to down-regulate DMN activity compared to controls, associating abnormal DMN activity with depressive rumination [94, 95]. Our patient group indeed showed significantly greater depression scores compared with controls (see Table 1).

SUD involves compulsive pursuit of the drug along with craving. Such characteristics have been hypothesized to be related to OFC and its strong connections with limbic and reward pathways [84]. Individuals with lesions to ACC/OFC may exhibit externalizing behaviors, including problems of inhibition and poor behavioral control [84], problems often seen in youths with or at risk for SUD [3, 16, 20]. Patients like ours may display emotional dysregulation [96], problems of executive control and inhibition [29], impulsiveness [20, 97], problems with error processing, and difficulty learning from punishment [98]. ACC and OFC dysfunction can be reasonably related to all of these traits.

We attempted to test empirically whether our patient-control cortical thickness difference in left pregenual RACC-mOFC was better explained by problems of affect regulation or externalizing behavior problems. However, we found that controlling for both depression severity and severity of externalizing behavior problems eliminated the patient-control difference. While this result is not simple or straightforward and does not link one co-morbidity to our brain finding, it suggests that risk for SUD in female adolescents may be complex, implicating both depression and externalizing problems.

### Sex Differences from Secondary Analyses

We unexpectedly found no differences in cortical thickness between our prior male and current female samples (see Results, Secondary Analyses). Although sex differences have been demonstrated in regions relevant to the current study (e.g., medial oribitofrontal cortex) [99,100], sex differences in cortical thickness appear much less prominent than sex differences in cortical volume and surface area [100]. Males and females differ in cortical thickness developmental trajectories [99] but in many instances those trajectories appear to decussate in the adolescent years (see Figure 4 in publication [99]). For these reasons, detection of sex differences for cortical thickness in SUD populations may require larger samples than those utilized here and perhaps a focus on younger or older populations; in other words, the lack of sex differences demonstrated here may be because of limited power and our adolescent focus. These results might support that future studies focusing on brain cortical thickness in this population might consider studying males and females together. However, we would suggest caution in this approach. Our male and female patients differed significantly in the prevalence of conduct disorder. Thus, as expected from the extant literature, the pattern of co-morbidity in adolescent males and females with SUD differed and prior authors suggest important potential phenotypic differences in males and females (see Introduction, A Female-Only Sample). Studying females specifically allowed examining the unique contributions of internalizing and externalizing scores on our cortical thickness finding. As mentioned above, our analyses may have missed important but smaller male:female differences in cortical thickness due to our modest sample sizes (see Discussion, Limitations).

### The Meaning of Cortical Thinning in Relation to Function

Although we demonstrate that patients compared to controls had thinner cortex in a portion of the RACC/OFC, it is not clear what the functional relevance of such “thinning” represents. There are certainly normative age related changes in cortical thickness, including both synaptogenesis and then thinning in the adolescent years. That thinning is hypothesized to be related to important maturational changes that improve synaptic efficiency such as increased myelination and synaptic pruning [101, 102]. Therefore, in some instances cortical thinning has been associated with improved function, (e.g., with improved general verbal intellectual functioning; [103]), and failure of normative cortical thinning has also been linked to problems of emotional control and behavioral regulation [104]. In many other instances, cortical thinning is associated with neurological decline [105–107]. Differences seen here could relate to developmental differences in synaptogenesis, early or more extensive synaptic pruning, substance-related injury, among other possible explanations. Longitudinal imaging designs of this important population are needed to better place such findings in a developmental context.

### ROI vs. Whole Brain Results

It is important to note that while our whole brain analyses demonstrated patient-control differences in left RACC/OFC, our region of interest analyses (which also looked at ACC and OFC) did not yield significant group differences. While this appears contradictory on first blush, the two approaches have many important differences. Our whole brain analyses search for areas of group difference where each vertex differs in cortical thickness (at a vertex-level p<0.005) while also requiring a Monte-Carlo-simulation-determined minimum number of contiguous vertices meeting the vertex-level requirement (250 mm^2^). This approach allows us to find smaller focal regions of greater patient-control differences and allows identified brain regions of group differences to cross boundaries (e.g. different Brodmann Areas or different gyri). Our region of interest analyses test for group differences in average cortical thickness across larger pre-defined brain regions. This approach, compared to our whole brain analyses, can identify less extreme patient-control differences in cortical thickness (p<0.05 at the ROI level vs. p<0.005 at each vertex) within these regions. Hence, the two approaches are complementary.

### Regression Analyses and Cortical Thickness

We completed regression analyses between cortical thickness with recency of drug use and separately with severity of externalizing behavior problems (BD scores) on a whole-brain vertex-level and within the left RACC-mOFC region identified in our primary patient-control comparisons. The purpose of this was to examine whether there was a significant component of cortical thinning secondary to direct substance effects that recovered with abstinence. Alternatively, we expected a correlation between BD severity and cortical thinning in the setting of predisposing brain differences. The utilization of BD scores, a measure that has been shown in the past to effectively capture a strongly heritable component of externalizing behaviors, allows us to propose the potential meaning of this correlation [28].

In performing these analyses at the whole-brain level we demonstrate no association with recency of use and one cluster showing a positive correlation between BD severity and cortical thickness within the left precuneus. This region is considered as the “functional core” of the DMN playing a “pivotal role” in the appropriate functioning of the network [108, 109]. Prior work has found increased precuneus connectivity with DMN regions in depressed subjects [110], which aligns with the hypothesis that our findings in our adolescent female population are associated with problems of affective control. We see both increased cortical thickness in the precuneus (in association with BD) and decreased thickness in the pregenual RACC-mOFC (between groups). Both are regions critical to the DMN, suggesting this network may be important to understanding patient-control differences in SUD risk in adolescent females.

### Strengths and Limitations

This study provides the opportunity to add to our understanding of brain morphometry related specifically to SUD in adolescent females. Studying adolescents conveys some advantages. These adolescent patients have substance problems severe enough to merit treatment entry early in life (see Table 1). But unlike adult studies of samples with many more years of chronic substance exposure, these youths had relatively few years of heavy substance exposure (see bottom row Table 1); if the brain differences identified in this study are substance induced, such brain changes occur with relatively few years of heavy exposure in adolescence. Studying only females is another strength of this work. As highlighted in section 1.2, there is mounting evidence of important sex differences at the phenotypic level that may play a role in SUDs.

Our study also has several limitations. To our knowledge, this is one of the first studies testing differences in cortical thickness in SUD female youths, but our sample of 22 patients and 21 controls may have relatively modest power to detect whole-brain cortical thickness differences [111]. For example, Pardoe and colleagues [111] estimate under certain assumptions (e.g., alpha 0.05, two-sided test, 10mm surface-based smoothing, etc.) that 20 subjects per group has 80% power to detect mean cortical thickness differences of about 0.4–0.5 mm, with some variability based on lobe of interest. Therefore, regions with modest between-group differences may not have been identified in our current analyses. Future studies with larger samples will reduce these concerns. Also, we study female adolescents, and our results should not be extrapolated to male adolescents or to adults with serious substance problems. Additionally, although we describe less cortical thickness in patients versus controls, the functional relevance of such cortical thinning is not fully understood (as described in Section 4.3). Finally, as is apparent from our inclusion/exclusion criteria, we did not clean our sample of comorbid mental health concerns, broadly including female adolescents with SUD. By taking this “broad” approach, we are able to recruit a sample that is more representative of the treatment population of interest. Given that SUD, externalizing behavior problems, as well as problems of impulsivity and/or high novelty seeking tend to cluster within individuals [65, 97] in a highly heritable fashion [11, 14, 19, 28], removing all comorbidity would lead to an atypical, less severely affected sample [112]. Nevertheless, our “broad” approach does result in some limitations, most notably that we cannot assess the contributions of specific diagnosis to a specific finding. However, our “broad” approach provides complementary information to studies employing a “narrow” strategy to recruit subjects with a single SUD and no other co-morbid disorders.

### Future Directions

Various studies of brain morphometry in SUD youths, or similar phenotypes, now suggest either cortical thinning [48, 113–117] or less grey matter volume [35, 37, 38] in such youths, with some important exceptions [118]. Many of these studies further implicate various frontal regions important to decision-making and executive control. Unfortunately, no brain morphometric changes appear clearly pathognomonic. Future studies could benefit from larger sample sizes to identify group differences with smaller effect sizes. Longitudinal designs may also better separate predisposing from substance-induced changes and identify patient-control differences in developmental trajectories. If clear and replicable brain differences associated with SUD are identified, approaches such as transcranial direct current stimulation might be employed to test potential mitigation of such behavior problems.

## Supporting Information

S1 Table Comparing Inclusion and Exclusion Criteria for Our Female Sample with Male Sample Published Previously. (DOCX)

S2 Table Comparing Males and Females Within Patients and Within Controls for Demographics and Key Clinical Measures. (DOCX)

S1 Text Testing the Effects of Edits. (DOCX)
