# Supplementary material for: Brain Cortical Thickness Differences in Adolescent Females with Substance Use Disorders
Source: PLoS One. 2016 Apr 6;11(4):e0152983. doi: 10.1371/journal.pone.0152983 (PMC4822952; doi:10.1371/journal.pone.0152983)
Supplement: S1 Table — (DOCX) [file pone.0152983.s001.docx]

S1 Table. Comparing Inclusion and Exclusion Criteria for Our Female Sample with Male Sample Published Previously

| All | Female Patients | Male Patients | Female Controls | Male Controls |
| --- | --- | --- | --- | --- |
| Inclusion Criteria | | | | |
|  | - Age 14-19 - Enrolled in University-based treatment program - Meets criteria for at least one non-nicotine DSM-IV-TR substance abuse or dependence diagnosis | - Age 14-18 - Enrolled in University-based treatment program - Meets criteria for at least one non-nicotine DSM-IV-TR substance abuse or dependence diagnosis | - Age 14-19 | - Age 14-18 |
| Exclusion Criteria | | | | |
| - Standard MRI criteria for adolescents - Failure to provide negative urine (drug) & saliva (alcohol) tests 7 days and immediately prior to scanning - History of serious neurological illness - Prior neurosurgery - History of unconsciousness lasting greater than 15 minutes - Color blindness - High risk of suicide, psychosis, violence, or fire setting - Estimated IQ < 80 - Left-handedness | - Positive pregnancy test |  | - Previous court conviction - Substance related arrest, treatment, or school expulsions - Meeting DSM-IV-TR criteria for a non-nicotine substance abuse or dependence diagnosis - Meeting DSM-IV-TR criteria for conduct disorder in the last year - Positive pregnancy test | - Previous court conviction - Substance related arrest, treatment, or school expulsions - Meeting DSM-IV-TR criteria for a non-nicotine substance abuse or dependence diagnosis - Meeting DSM-IV-TR criteria for conduct disorder in the last year |

Abbreviations: DSM-IV-TR = Diagnostic and Statistical Manual of Mental Disorders, Fourth Edition, Text Revision; Estimated IQ = intelligence quotient estimated using the vocabulary and matrix reasoning subtests of the Wechsler Abbreviated Scale of Intelligence
