# Supplementary material for: Brain Cortical Thickness Differences in Adolescent Females with Substance Use Disorders
Source: PLoS One. 2016 Apr 6;11(4):e0152983. doi: 10.1371/journal.pone.0152983 (PMC4822952; doi:10.1371/journal.pone.0152983)
Supplement: S2 Table — (DOCX) [file pone.0152983.s002.docx]

S2 Table. Comparing Males and Females Within Patients (n=47) and Within Controls (n=40) for Demographics and Key Clinical Measures.

|  |  | Female Patients (n=22) | Male Patients (n=25) | Test Statistic; p-value | Female Controls (n=21) | Male Controls (n=19) | Test Statistic; p-value |
| --- | --- | --- | --- | --- | --- | --- | --- |
| Age in years mean (SEM) |  | 16.1 (0.20) | 16.6 (0.23) | t_45_=1.81; p=0.08 | 16.7 (0.25) | 16.6 (0.37) | t_32.15_=-0.19; o=0.85 |
| Race n (%) | Caucasian | 12 (54.5) | 16 (64.0) | Χ^2^_1_=0.43; p=0.51 | 13 (61.9) | 15 (78.9) | Χ^2^_1_=1.38; p=0.24 |
| Estimate IQ mean (SEM) |  | 94.3 (2.23) | 98.1 (1.68) | t_45_=1.35; p=0.18 | 104.0 (2.26) | 105.2 (2.08) | t_38_=0.41; p=0.69 |
| CD lifetime diagnosis n (%) |  | 14 (63.6) | 22 (88.0) | **Χ^2^_1_=3.88; p=0.049** | 0 | 0 | NA |
| Aggression mean (SEM) |  | 5.7 (0.55) | 5.7 (0.62) | t_45_=-0.06; p=0.96 | 0 (0) | 0.4 (0.23) | MW p=0.41 |
| Impulsivity mean (SEM) |  | 14.7 (1.23) | 12.8 (1.23) | t_45_=-1.10; p=0.28) | 5.6 (1.00) | 7.0 (1.04) | t_38_=0.92; p=0.36 |
| Depression mean (SEM) |  | 11.0 (1.23) | 8.8 (1.21) | t_45_=-1.27; p=0.21 | 4.3 (0.77) | 4.2 (0.90) | t_38_=-0.15; p=0.88 |
| SUMDEP  mean (SEM) |  | 13.1 (1.66) | 11.9 (1.41) | t_45_=-0.56; p=0.58 | 0.2 (0.24) | 0.2 (0.16) | MW p>0.99 |
| ADHD t-score mean (SEM) |  | 60.7 (1.56) | 56.8 (1.70) | t_45_=-1.71; p=0.10 | 52.9 (1.03) | 53.2 (1.24) | MW p=0.96 |

Abbreviations: CD = conduct disorder; Combined ADHD t-score = DSM-IV-TR defined attention-deficit/hyperactivity disorder t scores measured using the Youth Self Report; Depression= Carroll Rating Scale for depression score; estimated IQ = intelligence quotient estimated using the vocabulary and matrix reasoning subtests of the Wechsler Abbreviated Scale of Intelligence; MW = Mann-Whitney U test; SEM = standard error of the mean; SUMDEP = total number of substance dependence symptoms across 10 drug categories.
