# Supplementary material for: Brain Cortical Thickness Differences in Adolescent Females with Substance Use Disorders
Source: PLoS One. 2016 Apr 6;11(4):e0152983. doi: 10.1371/journal.pone.0152983 (PMC4822952; doi:10.1371/journal.pone.0152983)
Supplement: S1 Text — (DOCX) [file pone.0152983.s003.docx]

**S1 Text: Testing the Effects of Edits**

To assure that manual image editing in the FreeSurfer software had not produced false positive group differences, despite the editor being blinded to group assignment, we reran group comparisons in QDEC on the unedited data. We also tested whether editing caused greater changes of ROI cortical thickness in one group vs. the other. When whole-brain analysis was performed on the unedited data patient-control differences in the pregenual rostral anterior cingulate extending into the medial orbitofrontal cortex remained significant and were larger (cluster size = 389.13 mm^2^). Editing did not cause a greater change in cortical thickness for patients compared to controls for any ROI examined. Change in cortical thickness (cortical thickness of the ROI without editing minus the thickness of the ROI after editing) for each ROI was: Left-mOFC (Pt mean=-0.031; Ct mean=-0.022; p=0.76), Right-mOFC (Pt mean=-0.028; Ct mean=-0.023; p=0.88), Left-RACC (Pt mean=-0.012; Ct mean=0.023; p=0.42), Right-RACC (Pt mean=0.009; Ct mean=0.019; p=0.79), Left-MFG (Pt mean=-0.013; Ct mean=-0.007; p=0.76), and Right-MFG (Pt mean=0.450; Ct mean 0.490; p=0.18).
